# Transcriptome sequencing revealed molecular mechanisms underlying tolerance of *Suaeda salsa* to saline stress

**DOI:** 10.1371/journal.pone.0219979

**Published:** 2019-07-23

**Authors:** Su-Ming Guo, Ying Tan, Han-Jie Chu, Mei-Xia Sun, Jin-Cheng Xing

**Affiliations:** 1 College of Landscape Architecture, Nanjing Forestry University, Nanjing City, Jiangsu Province, P. R. China; 2 College of Architecture, Southeast University, Nanjing City, Jiangsu Province, P. R. China; 3 Jiangsu Coastal Area Institute of Agricultural Sciences, Yancheng City, Jiangsu Province, P. R. China; ICAR-Indian Institute of Agricultural Biotechnology, INDIA

## Abstract

The halophyte *Suaeda salsa* displayed strong resistance to salinity. Up to date, molecular mechanisms underlying tolerance of *S*. *salsa* to salinity have not been well understood. In the present study, *S*. *salsa* seedlings were treated with 30‰ salinity and then leaves and roots were subjected to Illumina sequencing. Compared with the control, 68,599 and 77,250 unigenes were significantly differentially expressed in leaves and roots in saline treatment, respectively. KEGG enrichment analyses indicated that photosynthesis process, carbohydrate, lipid and amino acid metabolisms were all downregulated in saline treatment, which should inhibit growth of *S*. *salsa*. Expression levels of Na^+^/H^+^ exchanger, V-H^+^ ATPase, choline monooxygenase, potassium and chloride channels were upregulated in saline treatment, which could relieve reduce over-accumulation of Na^+^ and Cl^-^. Fe-SOD, glutathione, L-ascorbate and flavonoids function as antioxidants in plants. Genes in relation to them were all upregulated, suggesting that *S*. *salsa* initiated various antioxidant mechanisms to tolerate high salinity. Besides, plant hormones, especially auxin, ethylene and jasmonic acid signaling transduction pathways were all upregulated in response to saline treatment, which were important to gene regulations of ion transportation and antioxidation. These changes might comprehensively contribute to tolerance of *S*. *salsa* to salinity. Overall, the present study provided new insights to understand the mechanisms underlying tolerance to salinity in halophytes.

## Introduction

The halophyte *Suaeda salsa* (L.) Pall is a leaf succulent annual plant. It is widely distributed in Eurasia and has been cultivated as a seawater vegetable in desert and coastal areas in P. R. China [1]. *S*. *salsa* shows strong tolerance to salinity. In comparison to the control, treatments with moderate salinities (varied from 50 mM to 200 mM NaCl) promoted CO_2_ assimilation rate, O_2_ production, photosynthesis rate and thus plant growth of *S*. *salsa* [2–6]. However, high salinities (> 200 mM NaCl) inhibited its growth significantly [6], displaying much higher tolerance to saline stress than other plants.

*S*. *salsa* should have special mechanisms underlying tolerance to high salinity. Physiologically, differential regulation of abscisic acid (ABA), gibberellins (GA) homeostasis [7] and Na^+^ storage [6] might provide strategies for *S*. *salsa* plants to survive adverse environmental conditions. Accumulation of proline and decreased content of soluble sugars in leaves promoted osmotic adaptation to saline stress [6]. Molecularly, functions of several genes were characterized. For example, glycerol-3-phosphate acyltransferase (GPAT) [8] and vacuolar H^+^/Ca^2+^ transporter [9] enhanced saline tolerance. Activity of plasma membrane aquaporins (AQPs) is involved in leaf succulence in *S*. *salsa* and also regulates its tolerance to salinity [10]. Besides, gene expression levels of myo-inositol-1-phosphate synthase (INPS), choline monooxygenase (CMO), betaine aldehyde dehydrogenase (BADH), catalase (CAT), and activities of superoxide dismutase (SOD), peroxidase (POD), CAT, and glutathione peroxidase (GPx) were elevated when *S*. *salsa* was treated with salinity, suggesting that these genes/enzymes might also contribute to saline tolerance in *S*. *salsa* [11]. Moreover, metabolic profiling revealed that saline treatments depleted contents amino acids, malate, fumarate, choline, phosphocholine, and elevated betaine and allantoin in *S*. *salsa* seedlings as well as reduced contents of glucose and fructose but increased contents of proline, citrate, and sucrose in roots [11]. These results revealed molecular mechanisms underlying saline tolerance in *S*. *salsa*. However, comprehensive profiles were still lacking.

In other halophytes or model plant species, studies have reported some molecular mechanisms underlying saline tolerance. In response to saline treatment, expansins, phosphatase, ethylene-related pathways were upregulated in *Suaeda glauca* and *Suaeda maritima* [12,13]. WRKY transcription factors involved in drought and saline tolerance were upregulated in saline treatments in *S*. *glauca* [12], *Nicotiana benthamiana* [14], *Populus tomentosa* [15], *Jatropha curcas* [16] and *Arabidopsis thaliana* [17]. Choline monooxygenase (CMO) and betaine aldehyde dehydrogenase (BADH) contributed to saline resistance in *S*. *maritima* [13,18] and rice [19]. Fatty acid desaturase was required for *A*. *thaliana* [20], *S*. *glauca* [12] and tomato [21] to resist saline stress. Expression levels of cytochrome P450s increased in salt-treated *S*. *glauca* [12], *Gossypium hirsutum* and *Phaseolus vulgaris* [22]. MYB genes were elevated in sweet cherry [23], *Salicornia brachiate* [24] and S. maritima in response to saline treatments [13]. There results indicated that different plants may adopt different molecular strategies to resist saline stress.

Transcriptome sequencing is a powerful tool to explore molecular mechanisms underlying biological process. To the best of our knowledge, no reports have been reported to investigate changes of transcripts of *S*. *salsa* in response to saline stress. In the present study, *S*. *salsa* seedlings were treated with 30‰ salinity and then leaves and roots were subjected to transcriptome sequencing. Real-time quantitative PCR (qPCR) was performed to validate the results. Bioinformatics analyses were adopted to compare changes at mRNA and pathway levels. These data would be helpful to clarify the molecular mechanisms underlying tolerance of *S*. *salsa* to salinity.

## Materials and methods

### Ethics statement

No specific permit is required for studies on *S*. *salsa* in P. R. China. During the experiments, no local regulations or laws were overlooked.

### Germination of *S*. *salsa* seeds in response to saline treatments

*S*. *salsa* seeds were kindly donated by Jiangsu Coastal Area Institute of Agricultural Sciences (Yancheng, P. R. China). Six saline concentrations were prepared, including 0.94‰, 1.88‰, 3.75‰, 7.5‰, 15‰ and 30‰ (geometric sequence by two times), by dissolving commercial sea salts (Tangfeng Slats Company Limited, Tangshan, P. R. China) in deionized water. Deionized water was used as the control. Germination assays were conducted in 9 cm Petri dishes. In each dish, three layers of filter paper were placed at the bottom, 10 ml of saline solution was added and then 100 seeds were placed on filter paper. Seeds were germinated in a light humidified incubator at humidity of 80% RH and dark. The temperature was 25°C at day time (12 h) and 15°C at night (12 h). Germinated seeds were counted daily for 10 days to calculate germination rate. To avoid disturbance of evaporation on salinity, germinated seedlings were discarded and ungerminated seeds were transferred to new dishes with fresh solution every two days. Each assay was repeated four times.

### Saline treatments of *S*. *salsa* seedlings

*S*. *salsa* seeds were germinated in deionized water as described above. When seedlings reached 10 cm approximately, seedlings were transplanted to plastic containers with sands as cultural substrate and Hoagland’s solution as media, and then cultured in a greenhouse at 25°C. The light cycle was 16 hours: 8 hours (light: dark) and light intensity was approximately 14,400 lux. After two weeks, healthy plants were treated with salinity of 30‰ by dissolving commercial sea salts (Tangfeng Slats Company Limited, Tangshan, China) in Hoagland’s solution. Hoagland’s solution was used as the control. Each treatment included 20–30 individuals and repeated three times independently. To supplement evaporated water, distilled water was added every day to keep total volume of culture media consistent. After 10 days, plants were harvested. Roots and top four leaves were collected and immediately frozen in liquid nitrogen for RNA extraction. For each treatment, samples from 5 individuals were pooled.

### Transcriptome sequencing

Total RNA was extracted using Biozol reagent (Bioer, Hangzhou, China) according to the manufacture’s protocol. Quality of total RNA was check by 1% agarose gel electrophoresis, NanoPhotometer spectrophotometer (IMPLEN, CA, USA) and Agilent Bioanalyzer 2100 system (Agilent Technologies, CA, USA). RNA samples with RNA integrity number (RIN) higher than 8.0 were considered qualified. RNA concentration was measured using Qubit RNA assay kit on Qubit 2.0 Flurometer (Life Technologies, CA, USA).

To construct sequencing libraries, mRNA was enriched using NEBNext Poly(A) mRNA Magnetic Isolation Module (NEB, USA). Sequencing libraries were prepared using NEBNext mRNA Library Prep Master Mix Set for Illumina (NEB, USA) and NEBNext Multiplex Oligos for Illumina (NEB, USA). Sequencing library was viewed on 1.8% agarose gel to check insert size and quantified using Library Quantification Kit-Illumina GA Universal Kit (Kapa, USA).

Qualified libraries were clustered on a cBot cluster generation system using HiSeq 4000 PE cluster kit (Illumina) and sequenced on an Illumina Hiseq 2500 platform. Three independent samples were sequenced as three biological replicates.

### Bioinformatics analyses

Clean reads were achieved by removing adaptors, reads with N ratio higher than 1% and low quality reads (with > 50% bases having Phred quality score ≤ 15). Clean reads were subjected to assembly of unigene library using Trinity v2.0.6 [25]. HTSwq v0.6.0 was applied to calculate FPKM values (expected number of fragments per kilobase of transcript sequence per millions base pairs sequenced) of each unigene. Relative expression levels of each gene were compared among different groups using DESeq2 R package. Fold change ≥ 2 and q value (adjusted P value) < 0.001 were considered significantly changed.

DEGs were mapped to Gene Ontology (GO) database and Kyoto Encyclopedia of Genes and Genomes database (KEGG) [26] for enrichment of GO categories and KEGG pathways using BLAST software [27].

### Real-time quantitative PCR

Results of Illumina sequencing were validated using real-time qPCR. cDNA was prepared using BioRT cDNA first strand synthesis kit (Bioer, Hangzhou, China) with oligo(dT) primer. qPCR was carried out using BioEasy master mix (Bioer, Hangzhou, China) on a Line Gene9600 Plus qPCR machine (Bioer, Hangzhou, China). DEGs and primers used for qPCR are listed in S1 Table. Transcriptional elongation factor-1 alpha was used as the internal control. Relative expression levels of each gene between saline treatment and the control were calculated using the typical 2^-ΔΔCt^ method [28]. Three biological replicates were included for each treatment and three technical repeats were performed for each sample.

## Results and discussion

### Effects of salinity on seed germination

Previously, most experiments employed NaCl to represent saline stress, which might misestimate the effects of other components in sea salts on seed germination and plant growth [29–30]. In the present study, we used sea salts to prepare saline solutions, which should be more similar to real environments.

**Fig 1:**
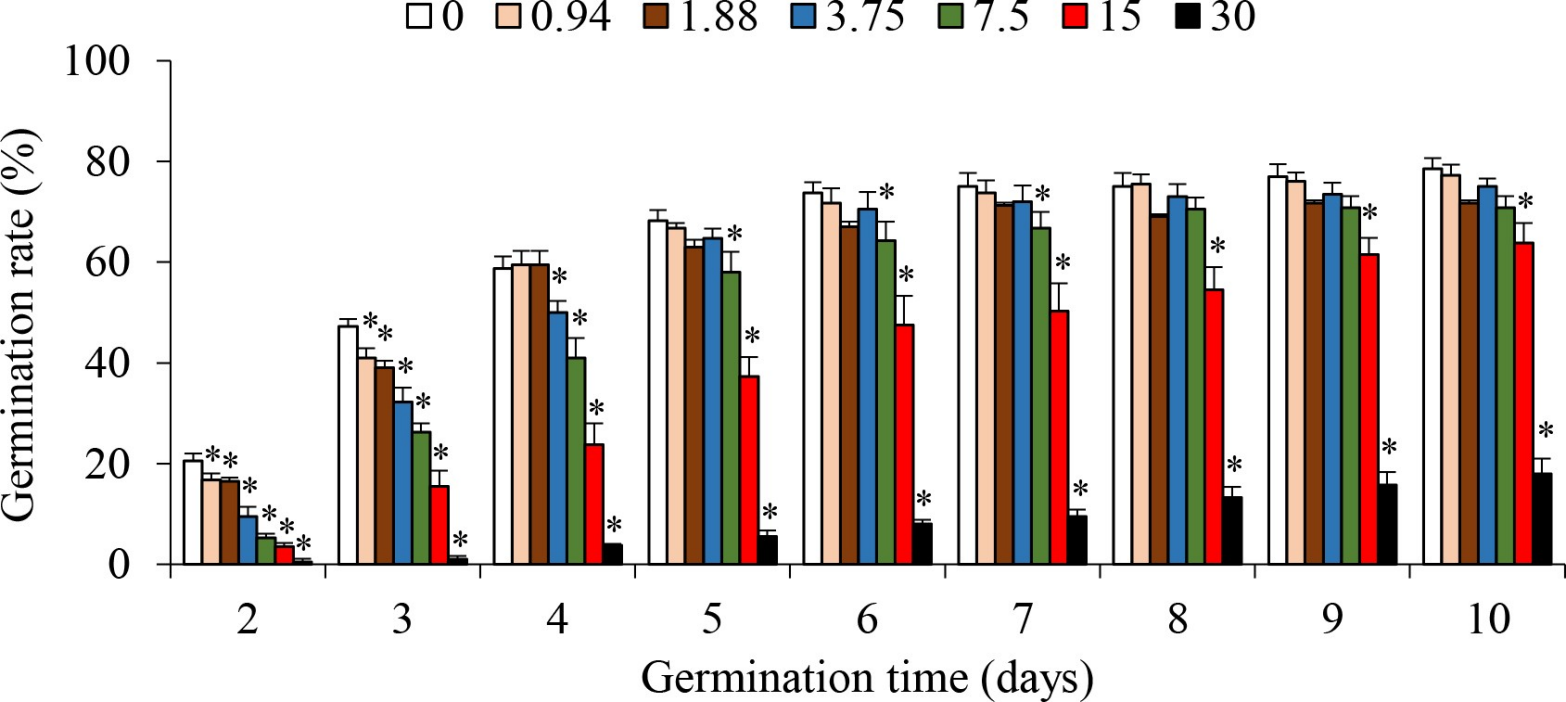
Effects of salinity on seed germination rate of *Suaeda salsa* (mean ± SE). * significantly different from the control (P < 0.05).

Seeds started to germinate in all treatments after two days. Germination rate significantly decreased in response to saline treatments but increased with germination time. After eight days, in comparison to the control, germination rates were significantly lower in treatments with 30‰ and 15‰, but did not significantly change in other saline treatments (Fig 1). These results suggested that germination of *S*. *salsa* seeds could tolerate salinity as high as 7.5‰, which was consistent with previous findings on *S*. *salsa* and the general consensus that germination of halophyte seeds is optimum under freshwater but decreases with elevating salinity [31, 32].

### Transcriptome sequencing

The sequencing data were deposited in NCBI with the reference number of PRJNA512222. Illumina sequencing resulted in 65.21 M to 72.28 M of total clean reads and 6.52 G to 7.23 G of total clean bases for each sample (Table 1). The sequencing depth was roughly calculated by the ratio of sequencing data to the size of assembled transcriptome, approximately ranging from 22.37 × to 24.81 ×. The total clean reads were higher than those in previous studies on *S*. *salsa* [33]. Besides, all Q20 and Q30 values were higher than 98.09% and 91.78%, respectively (Table 1). These indices suggested that the sequencing was deep and the as-obtained data should be enough for further analyses.

**Table 1:** Statistics of Illumina sequencing quality for each sample.

Salinity-Sample	TCR (M)	TCB (G)	Q20 (%)	Q30 (%)	TM (%)	UM (%)
Leaf						
Control-1	67.88	6.79	98.49	92.78	89.02	10.51
Control-2	67.52	6.75	98.39	92.42	89.51	10.62
Control-3	67.57	6.76	98.37	92.32	89.64	9.83
30‰-1	67.76	6.78	98.46	92.82	90.75	10.31
30‰-2	69.85	6.99	98.45	92.80	89.89	10.99
30‰-3	67.44	6.74	98.45	92.63	90.73	10.20
Root						
Control-1	70.26	7.03	98.52	92.91	87.74	12.26
Control-2	65.21	6.52	98.48	92.76	86.40	12.54
Control-3	66.91	6.69	98.42	92.58	86.83	12.83
30‰-1	70.19	7.02	98.09	91.78	87.04	12.11
30‰-2	72.28	7.23	98.15	91.88	87.00	12.53
30‰-3	67.59	6.76	98.15	91.91	88.23	12.36

TCR: total clean reads; TCB: total clean base; TM: total mapping; UM: uniquely mapping.

### *De novo* assembly

Total clean reads of each sample were assembled independently to get the unigene library of each sample. The total number of unigenes ranged from 40,038 to 133,430, with the mean length of unigenes from 798 bp to 1,180 bp (S2 Table). Benchmarking Universal Single-Copy Orthologs (BUSCO) analyses revealed that at least 2.97% BUSCOs were fragmented or missing (S1 Fig). Alternatively, clean reads of all samples were pooled, equal to 1,234.49 M of clean reads, and then subjected to Trinity assembly. Finally, 196,199 unigenes were obtained, with the mean length of 1,673 bp and the N50 length of 2,780 bp (Table 2). BUSCO analyses revealed that only 1.98% BUSCOs were missing and none BUSCOs were fragmented (S1 Fig). Obviously, the as-obtained unigenes showed longer mean length and N50 length as well as less fragmented and missing BUSCOs than those assembled from each sample (S1 Table), suggesting that unigene library generated from all samples had higher quality. The as-obtained unigene library was used for further analyses.

**Table 2:** Assembly statistics of unigenes and coding sequences (CDS).

Parameter	Unigene	CDS
Total clean reads used for assembly (M)	1,234.49	1,234.49
Total number	196,199	122,855
Total length (M bp)	328.25	137.21
Minimum unigene length (bp)	200	297
Maximum unigene length (bp)	35,045	15,345
Average unigene length (bp)	1,673	1,117
N50 length (bp)	2,780	1,431
N90 length (bp)	836	552
GC content (%)	39.23	42.78

### Functional annotation and analysis

Among the unigene library, more than 65.45% and 45.91%unigenes were longer than 500 bp and 1,000 bp, respectively (S2 Fig). Coding sequence (CDS) prediction revealed 122,855 CDSs, with the total length of 137.21 M bp, the average length of 1,117 bp and the N50 length of 1,431 bp (Table 2). Approximately 72.67% and 36.84% CDSs were longer than 500 bp and 1,000 bp, respectively (S2 Fig).

After blasted against the relevant databases, 61.97%, 51.39%, 43.88%, 47.03%, 48.15%, 44.30% and 14.91% unigenes could hit genes in the RefSeq non-redundant proteins (NR), nucleotide (NT), Swissprot, KEGG, Eukaryotic Orthologous Groups (KOG), Pfam and GO database respectively. Overall, 68.10% unigenes could be annotated to one or more databases.

### Differentially expressed genes and qPCR validation

**Fig 2:**
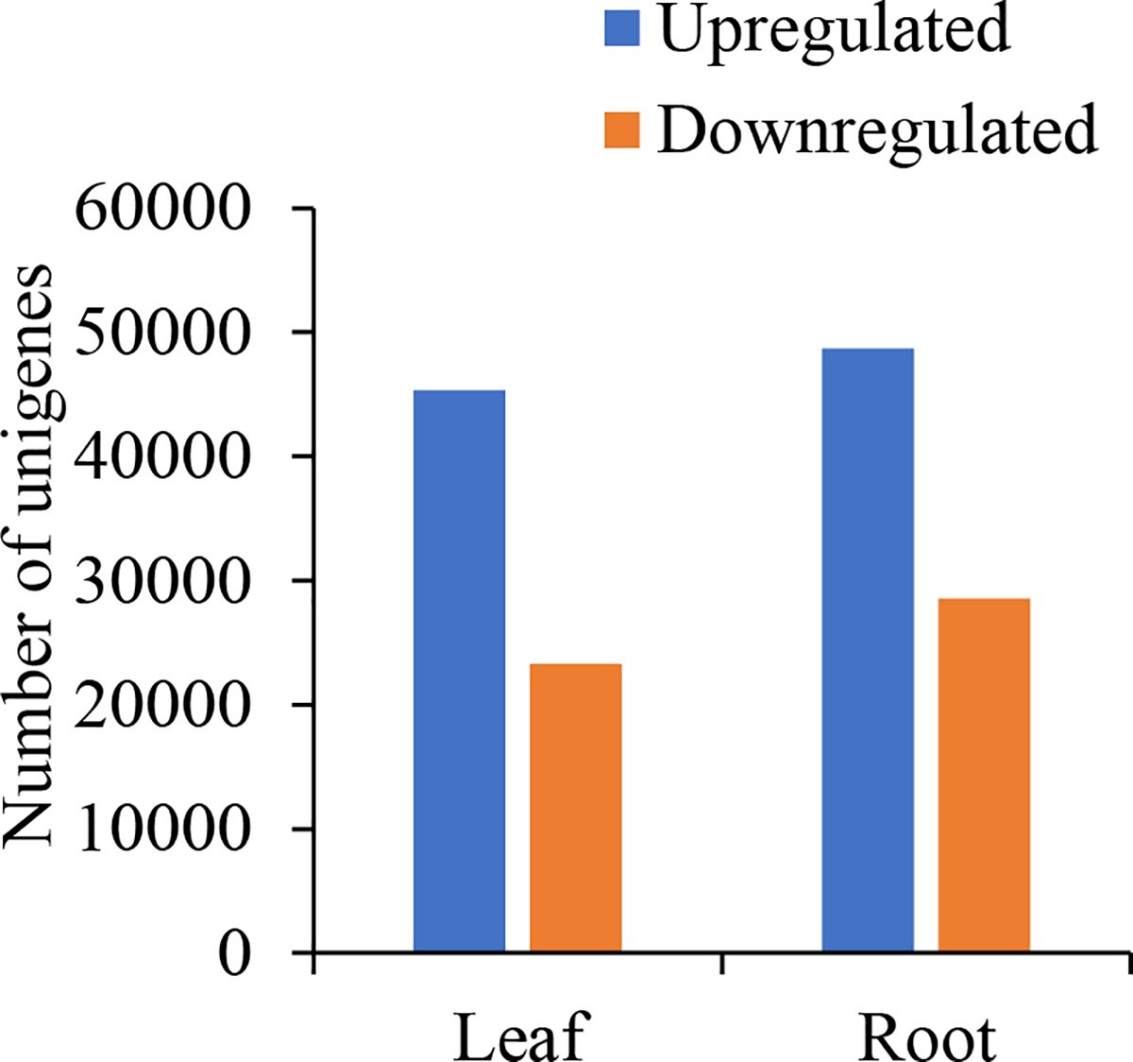
Number of differentially expressed genes in *Suaeda salsa* leaves and roots between treatment with 30‰ and the control.

Compared with the control, 68,599 (45,321 upregulated and 23,278 downregulated) and 77,250 unigenes (48,682 upregulated and 28,568 downregulated) were significantly differentially expressed in treatment with 30‰ in leaves and roots, respectively (Fig 2).

**Fig 3:**
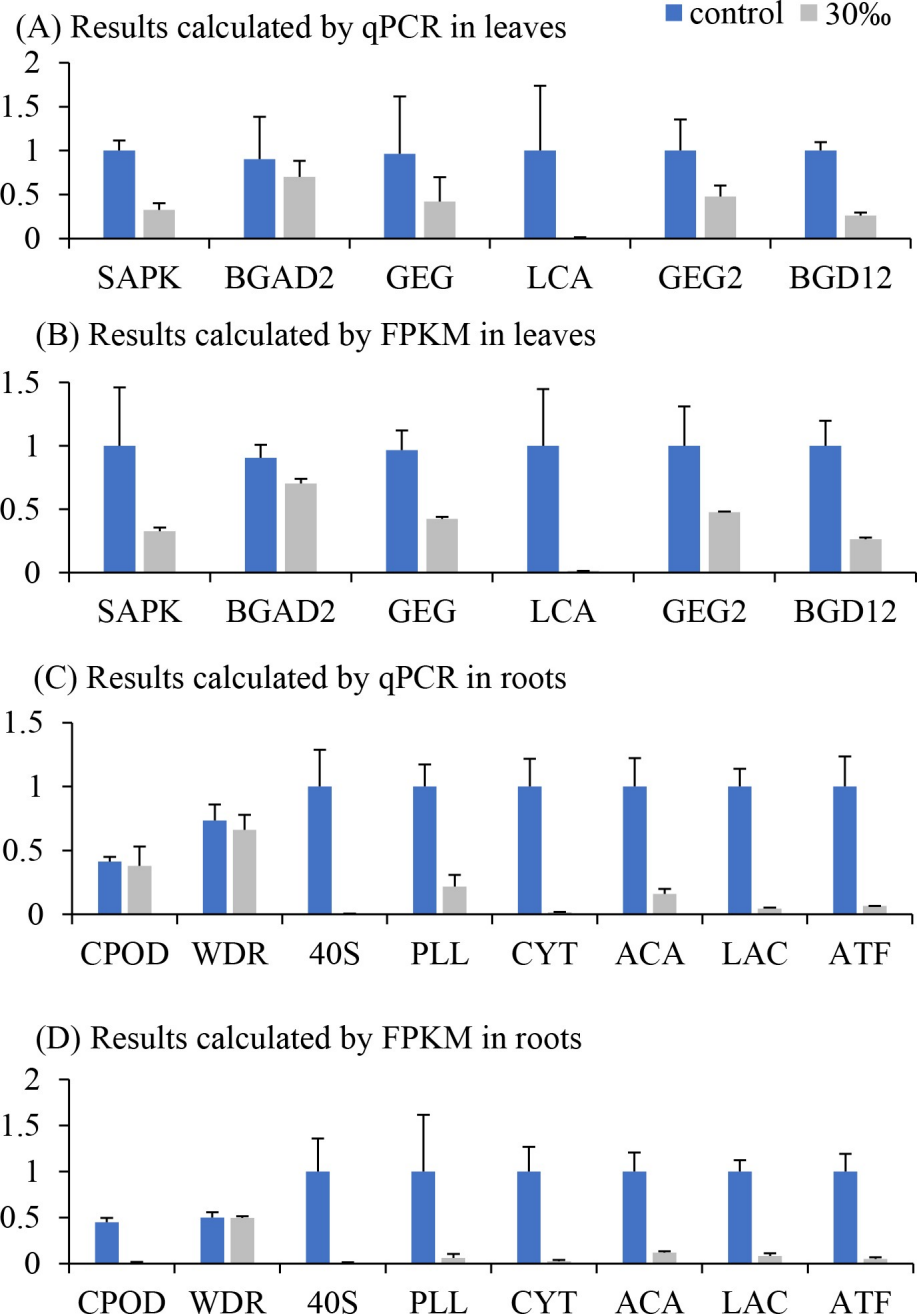
Real-time qPCR validation results (mean ± SE). Data were normalized by considering the highest value of each gene among three treatments as one. CPOD: Cationic peroxidase 1; WDR: WD repeat-containing protein 87; 40S: 40S ribosomal protein S3a; PLL: Probable linoleate 9S-lipoxygenase 5; CYT: Cytochrome P450 71A6; ACA: Acetyl-CoA acetyltransferase, cytosolic; LAC: Lipoamide acyltransferase component of branched-chain alpha-keto acid dehydrogenase complex, mitochondrial; ATF: Branched-chain-amino-acid aminotransferase 5, chloroplastic; SAPK: Serine/threonine-protein kinase; BGAD2: Beta-galactosidase 2; GEG: Glucan endo-1,3-beta-glucosidase; LCA: Lichenase; GEG2: Glucan endo-1,3-beta-glucosidase; BGD12: Beta-glucosidase 12.

To validate the expression levels predicted by transcriptome sequencing, qPCR was performed for 6 unigenes in leaves and 8 unigenes in roots. The results showed similar tendency to the expression levels calculated by FPKM value (Fig 3), suggesting that DEGs prediction was reliable.

### Enrichment of GO categories for DEGs

**Fig 4:**
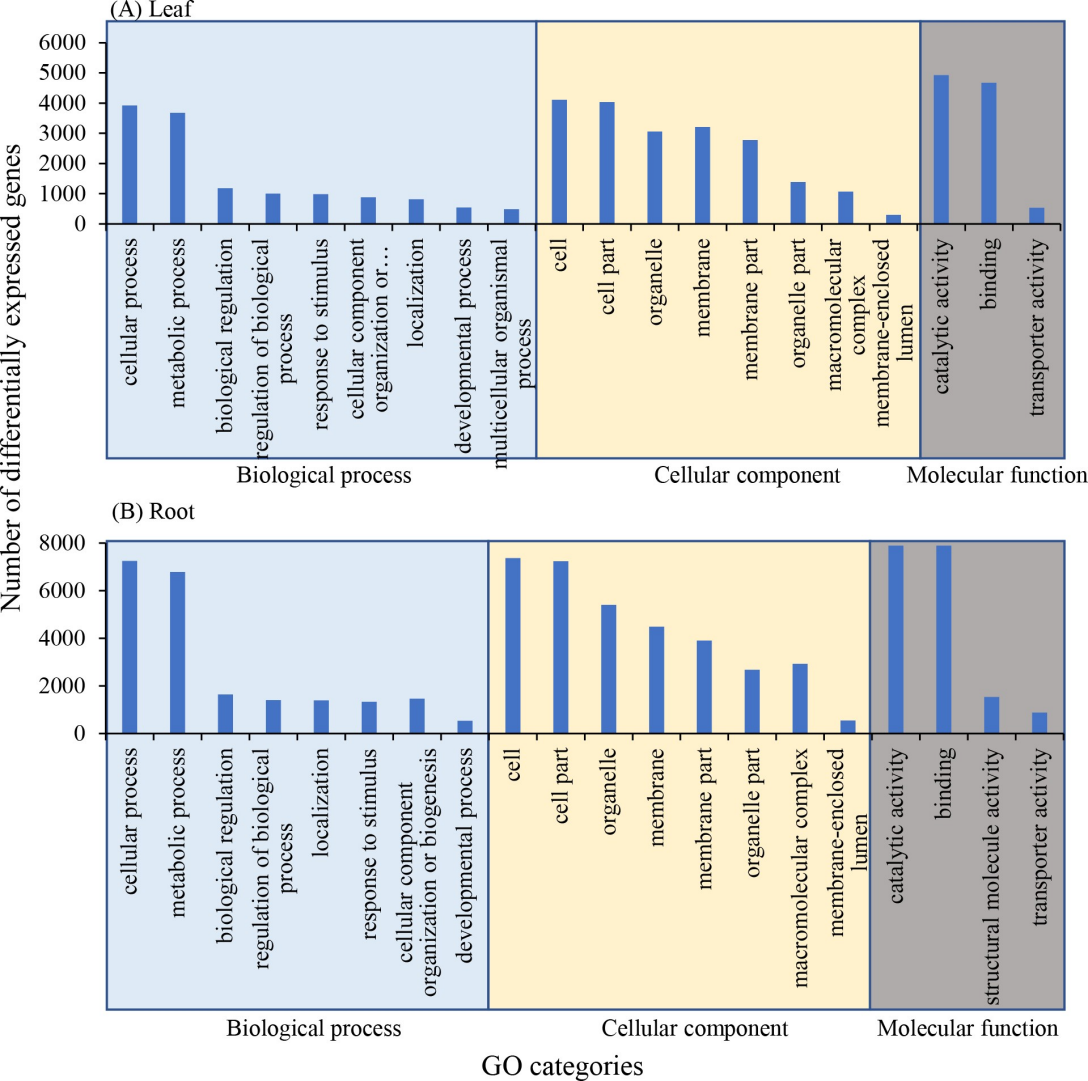
Top 20 GO categories of differentially expressed genes in treatment with 30‰ compared with the control.

Top 20 GO categories revealed that in leaves and roots, DEGs in comparison between 30‰ and control were mainly assigned to cellular process, metabolic process, cell, cell part, catalytic activity and binding (Fig 4).

### Enrichment of KEGG pathways for DEGs

KEGG enrichment of significantly downregulated genes showed 24 and 16 pathways in leaves and roots, respectively, which shared nine KEGG pathways, including valine, leucine and isoleucine degradation (ko00280), linoleic acid metabolism (ko00591), glycerophospholipid metabolism (ko00564), circadian rhythm-plant (ko04712), plant hormone signal transduction (ko04075), pentose phosphate pathway (ko00030), riboflavin metabolism (ko00740), nicotinate and nicotinamide metabolism (ko00760) and ascorbate and aldarate metabolism (ko00053). These enriched pathways represented harmful effects of salinity on *S*. *salsa*. KEGG enrichment of significantly upregulated genes revealed 17 and 18 pathways in leaves and roots, respectively. Between them, only one pathway (ko00261 monobactam biosynthesis) was shared. These results might provide new insights for investigation of molecular mechanisms underlying adaptation of *S*. *salsa* to salinity stress (Tables 3 and 4).

**Table 3:** Enrichment of KEGG pathway between treatment with 30‰ and the control in *Suaeda salsa* leaves. Enriched/total gene: number of DEGs/total genes in the corresponding pathway.

ID and name of KEGG pathway	Enriched/ total gene	P value	Q value
Upregulated genes in treatment with 30‰			
ko00480, Glutathione metabolism	361/1308	0.000	0.000
ko00901, Indole alkaloid biosynthesis	83/242	0.000	0.000
ko00940, Phenylpropanoid biosynthesis	550/2219	0.000	0.000
ko00130, Ubiquinone and other terpenoid-quinone biosynthesis	161/577	0.000	0.001
ko00941, Flavonoid biosynthesis	152/554	0.000	0.003
ko03018, RNA degradation	444/1866	0.001	0.017
ko04144, Endocytosis	621/2666	0.001	0.017
ko00300, Lysine biosynthesis	62/207	0.001	0.019
ko00460, Cyanoamino acid metabolism	228/914	0.002	0.021
ko00073, Cutin, suberine and wax biosynthesis	71/247	0.002	0.022
ko00261, Monobactam biosynthesis	58/195	0.002	0.022
ko00945, Stilbenoid, diarylheptanoid and gingerol biosynthesis	80/284	0.002	0.022
ko04075, Plant hormone signal transduction	529/2271	0.002	0.022
ko04120, Ubiquitin mediated proteolysis	423/1797	0.003	0.026
ko00966, Glucosinolate biosynthesis	29/85	0.003	0.027
ko00400, Phenylalanine, tyrosine and tryptophan biosynthesis	155/613	0.005	0.037
Downregulated genes in treatment with 30‰			
ko00500, Starch and sucrose metabolism	480/2481	0.000	0.000
ko00051, Fructose and mannose metabolism	153/687	0.000	0.000
ko00940, Phenylpropanoid biosynthesis	424/2219	0.000	0.000
ko04075, Plant hormone signal transduction	434/2271	0.000	0.000
ko00604, Glycosphingolipid biosynthesis-ganglio series	51/185	0.000	0.001
ko00941, Flavonoid biosynthesis	123/554	0.000	0.001
ko00740, Riboflavin metabolism	75/307	0.000	0.001
ko04016, MAPK signaling pathway—plant	424/2271	0.000	0.001
ko00945, Stilbenoid, diarylheptanoid and gingerol biosynthesis	69/284	0.000	0.002
ko00010, Glycolysis / Gluconeogenesis	261/1352	0.000	0.003
ko00760, Nicotinate and nicotinamide metabolism	73/311	0.000	0.003
ko04712, Circadian rhythm—plant	138/672	0.001	0.006
ko00564, Glycerophospholipid metabolism	204/1046	0.001	0.006
ko00591, Linoleic acid metabolism	67/299	0.002	0.012
ko04626, Plant-pathogen interaction	458/2562	0.002	0.012
ko00280, Valine, leucine and isoleucine degradation	190/990	0.002	0.014
ko00565, Ether lipid metabolism	73/340	0.003	0.022
ko00052, Galactose metabolism	218/1166	0.003	0.023
ko00531, Glycosaminoglycan degradation	61/277	0.004	0.023
ko00520, Amino sugar and nucleotide sugar metabolism	318/1760	0.004	0.025
ko00030, Pentose phosphate pathway	137/707	0.005	0.031
ko00053, Ascorbate and aldarate metabolism	193/1033	0.006	0.032
ko00710, Carbon fixation in photosynthetic organisms	152/800	0.007	0.039

**Table 4:** Enrichment of KEGG pathway between treatment with 30‰ and the control in *Suaeda salsa* roots. Enriched/total gene: number of DEGs/total genes in the corresponding pathway.

ID and name of KEGG pathway	Enriched/ total gene	P value	Q value
Upregulated genes in treatment with 30‰			
ko03010, Ribosome	1423/3320	0.000	0.000
ko00196, Photosynthesis—antenna proteins	28/43	0.000	0.000
ko00630, Glyoxylate and dicarboxylate metabolism	290/856	0.000	0.000
ko00260, Glycine, serine and threonine metabolism	261/789	0.000	0.000
ko01230, Biosynthesis of amino acids	913/3127	0.000	0.001
ko01200, Carbon metabolism	901/3111	0.000	0.002
ko00072, Synthesis and degradation of ketone bodies	42/101	0.000	0.009
ko00220, Arginine biosynthesis	174/539	0.001	0.011
ko00195, Photosynthesis	82/230	0.001	0.012
ko03008, Ribosome biogenesis in eukaryotes	385/1294	0.001	0.016
ko00710, Carbon fixation in photosynthetic organisms	246/800	0.001	0.017
ko00190, Oxidative phosphorylation	373/1263	0.002	0.023
ko03013, RNA transport	871/3088	0.002	0.023
ko04145, Phagosome	304/1015	0.002	0.023
ko00920, Sulfur metabolism	111/342	0.004	0.041
ko03020, RNA polymerase	331/1126	0.005	0.045
ko00261, Monobactam biosynthesis	67/195	0.006	0.047
ko00750, Vitamin B6 metabolism	55/156	0.007	0.050
Downregulated genes in treatment with 30‰			
ko00280, Valine, leucine and isoleucine degradation	308/990	0.000	0.000
ko00591, Linoleic acid metabolism	91/299	0.000	0.000
ko00071, Fatty acid degradation	169/643	0.000	0.000
ko00640, Propanoate metabolism	141/525	0.000	0.000
ko00592, alpha-Linolenic acid metabolism	145/547	0.000	0.000
ko00250, Alanine, aspartate and glutamate metabolism	192/775	0.000	0.001
ko00350, Tyrosine metabolism	152/597	0.000	0.001
ko00564, Glycerophospholipid metabolism	247/1046	0.000	0.002
ko04712, Circadian rhythm—plant	164/672	0.000	0.005
ko04075, Plant hormone signal transduction	493/2271	0.001	0.009
ko02010, ABC transporters	185/789	0.001	0.014
ko00030, Pentose phosphate pathway	167/707	0.001	0.015
ko00740, Riboflavin metabolism	79/307	0.002	0.023
ko00760, Nicotinate and nicotinamide metabolism	80/311	0.002	0.023
ko00053, Ascorbate and aldarate metabolism	231/1033	0.004	0.036
ko00511, Other glycan degradation	124/525	0.005	0.044

### Changes of energy metabolism in *S*. *salsa* upon saline treatment

Photosynthesis process is quite sensitive to saline stress. In response to saline treatments, photosynthesis process was inhibited in maize [34]and mulberry [35], which should negatively affect accumulation of organic matters and plant growth. Similarly, salinity also displayed negative effects on shoot length of *S*. *salsa* [36]. In the present study, carbon fixation in photosynthetic organisms (ko00710) was suppressed in saline-stressed leaves, suggesting that photosynthesis process might be inhibited by salinity, which then should reduce metabolism of organic carbons, displaying downregulation of carbohydrate metabolism (ko00500 starch and sucrose metabolism, ko00051 fructose and mannose metabolism, ko00010, glycolysis/gluconeogenesis, ko00052 galactose metabolism, ko00531 glycosaminoglycan degradation, ko00520 amino sugar and nucleotide sugar metabolism, ko00030 pentose phosphate pathway, ko00640 propanoate metabolism and ko00511 other glycan degradation). Furthermore, downregulation of carbohydrate metabolism would depress lipid metabolism (ko00564, glycerophospholipid metabolism, ko00591 linoleic acid metabolism, ko00071 fatty acid degradation, and ko00592 alpha-linolenic acid metabolism), amino acid metabolism (ko00250 alanine, aspartate and glutamate metabolism and ko00350 tyrosine metabolism) and biosynthesis of complicated polymer compounds (Tables 3 and 4). For example, phenylpropanoid biosynthesis (ko00940), which contributes monolignols, the starting compounds for biosynthesis of lignin [37], was downregulated in the present study. Overall, these changes would comprehensively depress *S*. *salsa* growth. These results were partially similar to transcriptomic analyses of *S*. *maritima* in treatment with 2% NaCl, which decreased the expression levels of genes related to photosynthesis (particularly the light reaction) and Calvin cycle, but increased most of the genes in the anabolic pathways (such as sucrose and starch synthesis) and catabolic pathways (such as the tricarboxylic acid cycle, glycolysis and the oxidative pentose phosphate pathway). The different changes of sucrose and starch synthesis and glycolysis between these two studies might be attributed to the tested salinities. In the present study, 30‰ salinity was used, which was much higher than that prepared by 2% NaCl.

The KEGG pathway riboflavin metabolism (ko00740) and nicotinate and nicotinamide metabolism (ko00760) is important for biosynthesis of vitamin B2 and B3, respectively. Vitamin B2 is an essential coenzyme to oxidordeuctases (such as succinodehydrogenase, xanthine oxidase and NADH dehydrogenase), participating in degradation of sugars. Vitamin B3 is a major component of coenzyme I (NAD) and coenzyme II (NADPH), which participate in the photosynthesis process, degradation of sugars and lipids. Thus, downregulation of these two pathways could be considered as normal regulation in response to saline-inhibited photosynthesis and accumulation of organic matters.

### Changes of ion transportation

To resist saline stress, the first strategy is to relieve harmful accumulation of Na^+^ and Cl^-^. In NaCl-treated *S*. *maritima*, plasma membrane H^+^ ATPase (PM-H^+^ ATPase), Na^+^/H^+^ exchanger, Na^+^/H^+^ antiporter, vacuolar-type H^+^ ATPase (V-H^+^ ATPase), betaine aldehyde dehydrogenase (BADH) and choline monooxygenase (CMO) were upregulated, which were involved in ionic adjustment [13]. Similarly, Na^+^ influx transporter and the tonoplast Na^+^/H^+^ antiporter were upregulated in *Suaeda fruticose* under saline condition [38]. In the present study, partially similar results were revealed (Table 5). Na^+^/H^+^ exchanger, V-H^+^ ATPase and CMO were significantly upregulated in leaves and roots. These results suggested that *S*. *salsa* might share similar mechanisms with other *Suaeda* species underlying ionic balance (Na^+^ and H^+^) in response to saline stress. The H^+^-ATPase generates an electrochemical membrane potential in the plasma membrane and Na^+^/H^+^ exchanger may transport Na^+^ out of cells via this H^+^ gradient [39,40]. However, PM-H^+^ ATPase and Na^+^/H^+^ antiporter did not change in response to saline treatment in the present study and also in *S*. *glauca* [12], indicating that different *Suaeda* species might differ in regulation of proteins responsible for ionic balance.

**Table 5:** FPKM values of selected genes in saline treatment and control in *Suaeda salsa*.

Gene name	Leaves		Roots	
	Control	30‰	Control	30‰
Antioxidant enzymes				
Choline monooxygenase	56.1 ± 22.2	272.3 ± 87.6*	85.0 ± 12.4	119.4 ± 10.4*
Copper chaperone for superoxide dismutase	20.6 ± 1.3	47.2 ± 3.9*	22.0 ± 5.3	35.6 ± 9.0
Superoxide dismutase [Cu-Zn]	87.5 ± 17.6	81.2 ± 4.3	113.9 ± 19.5	172.1 ± 22.0
Superoxide dismutase [Fe]	27.1 ± 9.5	91.8 ± 9.3*	12.1 ± 2.9	57.8 ± 3.8*
Ion transportation				
Cation/H(+) antiporter	11.7 ± 3.3	17.2 ± 2.6	32.1 ± 6.3	18.5 ± 4.8*
K(+) efflux antiporter	24.6 ± 0.8	23.2 ± 1.4	25.8 ± 6.6	24.6 ± 5.1
Sodium/hydrogen exchanger	28.9 ± 10.4	42.8 ± 4.9*	15.5 ± 5.9	36.8 ± 13.7*
Cadmium/zinc-transporting ATPase	2463.2 ± 173.7	3376.2 ± 438.5	3263.6 ± 294.8	4306.5 ± 1458.9
Calcium-transporting ATPase	59.2 ± 3.0	36.4 ± 2.1*	186.4 ± 25.2	107.9 ± 8.2*
Copper-transporting ATPase	47.3 ± 5.6	41.8 ± 4.1	54.9 ± 6.9	38.5 ± 17.1
Phospholipid-transporting ATPase	15.4 ± 5.2	13.4 ± 3.1	16.7 ± 9.3	11.8 ± 5.4
Plasma membrane ATPase	94.1 ± 13.9	65.5 ± 5.0	48.1 ± 13.8	17.1 ± 13.7
Calcium permeable stress-gated cation channel	156.8 ± 16.7	178.8 ± 85.7	120.8 ± 10.7	119.7 ± 15.3
Chloride channel protein	171.5 ± 11.1	223.7 ± 10.7*	70.4 ± 3.4	75.0 ± 10.5
Cyclic nucleotide-gated ion channel	34.1 ± 5.8	44.0 ± 7.0	23.3 ± 5.2	18.9 ± 8.0
S-type anion channel	15.6 ± 4.4	45.9 ± 7.5*	15.9 ± 2.2	21.8 ± 8.8
Mechanosensitive ion channel protein	51.5 ± 7.5	62.9 ± 8.0	32.1 ± 10.6	35.5 ± 6.5
Potassium channel	9709.0 ± 438.9	13596.4 ± 1125.4*	10829.4 ± 711.0	13792.0 ± 3692.7
Probable cyclic nucleotide-gated ion channel	41.2 ± 3.1	28.5 ± 1.8*	32.1 ± 4.7	24.3 ± 2.1
Two pore calcium channel protein	15.6 ± 5.1	26.3 ± 4.9	23.2 ± 1.1	27.0 ± 7.1
Two-pore potassium channel	38.8 ± 6.9	57.6 ± 5.1*	63.3 ± 20.9	61.1 ± 6.4
Choline monooxygenase	56.1 ± 22.2	272.3 ± 87.6*	85.0 ± 12.4	119.4 ± 10.4*
V-type proton ATPase	348.9 ± 38.8	704.5 ± 33.4*	201.4 ± 14.1	398.2 ± 28.0*
Indole alkaloid biosynthesis				
methylesterase	288.1±77.8	778.7±127.6*	395.8±172.1	599.5±236.8
GDSL esterase/lipase	179.9 ±49.2	223.0±22.7	371.3±162.2	417.0±187.4
Cell wall-associated genes				
O-acyltransferase WSD1	21.8 ± 9.6	20.3 ± 6.1	10.5 ± 0.6	10.6 ± 1.9
Laccase	13.4 ± 3.5	52.7 ± 10.6*	61.2 ± 19.2	121.5 ± 42.7
leucine-rich repeat extensin	126.8 ± 28.2	133.2 ± 8.8	132.8 ± 24.5	190.1 ± 18.3*
Expansin	427.7 ± 228.4	369.5 ± 39.1	349.6 ± 91.1	531.6 ± 207.4
Cellulose synthase	157.4 ± 4.3	348.3 ± 36.9*	119.5 ± 5.1	358.5 ± 179.1
Cellulose synthase interactive	54.8 ± 21.1	117.8 ± 9.2*	35.6 ± 9.1	91.7 ± 21.6*

Data represent mean ± standard deviation of FPKM values (n = 3).

* significantly different from the control for the same organ.

Besides, potassium channel, two-pore potassium channel, chloride channel protein, S-type anion channel were significantly upregulated in saline-treated leaves in the present study (Table 5). Similar results have been reported in *Zostera marina* in which various K^+^ channels and transporters showed higher uptake capacity of K^+^ in response to saline treatment than those in the control [41]. Maintenance of K^+^ supply is a crucial feature of salt tolerance, since transportation of K^+^ could effectively adjust osmotic pressure among subcellular compartments and cytosol caused by increased vacuolar Na^+^ concentration [42]. Activity of anion channel is corresponded to chloride channel protein [43]. Upregulation of chloride channel proteins could effectively reduce Cl^-^ accumulation and increase tolerance to salinity in plants [44].

### Activation of antioxidant mechanisms in response to saline treatment

Generally, saline treatments induce oxidative stress to plants [45,46]. The halophyte *S*. *salsa* should have some mechanisms to avoid detrimental effects of oxidative stress. In the present study, we examined the expression level of superoxide dismutase (SOD), which is the first antioxidant enzyme in response to environmental stress. The results revealed different patterns between two types of SOD. In comparison to the control, expression level of Fe-SOD increased significantly for 3.4 and 4.8 times in saline-treated leaves and roots, respectively, but expression level of Cu/Zn-SOD did not change significantly (Table 5). Consistently, saline treatment drove greater increase of expression level of Fe-SOD than that of Cu-Zn SOD in rice varieties [47]. Although Fe-SOD and Cu/Zn-SOD have similar catalyzing functions, but their amino acid sequences appear to be unrelated [48]. Thus, they may respond to oxidative stress triggered by different stress factors. More investigations are required to distinguish their biological functions.

Comparison between saline treatment and the control suggested that the KEGG pathway glutathione (GSH) metabolism (ko00480) was upregulated in *S*. *salsa* leaves. Although GSH functions in nutrient metabolism and regulation of cellular events (including gene expression, DNA and protein synthesis, cell proliferation and apoptosis, signal transduction, cytokine production and immune response, and protein glutathionylation), the major role of GSH occurs in the antioxidant defense [49]. In response to saline treatments, GSH metabolism was activated in reed [50] and *Arthrospira platensis* [51]. Thus, activation of GSH metabolism might also contribute antioxidant capacity to protect *S*. *salsa* from saline-induced oxidation.

GSH is biosynthesized from glutamate, cysteine, and glycine [49]. In roots, saline treatment upregulated the KEGG pathway glycine, serine and threonine metabolism (ko00260) but downregulated alanine, aspartate and glutamate metabolism (ko00250). How the changes of these two pathways affected GSH metabolism still required more investigations.

**Fig 5:**
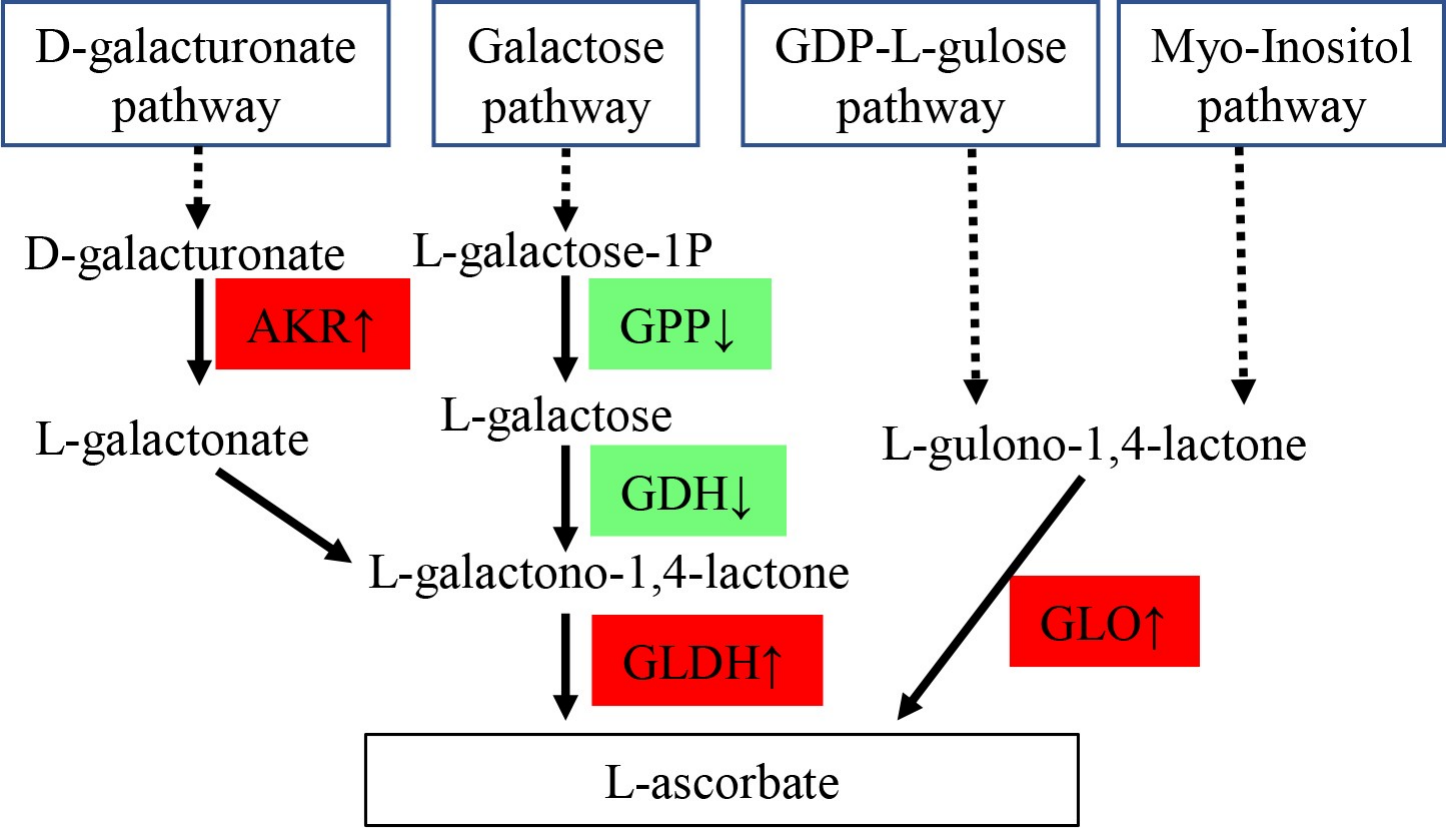
Changes of key genes in relation to L-ascorbate biosynthesis. AKR: aldo-keto reductase; GLDH: L-galactono-1,4-lactone dehydrogenase; GPP: L-galactose-1-phosphate phosphatase; GDH: L-galactose-1-dehydrogenase; GLDH: L-galactono-1,4-lactone dehydrogenase; GLO: L-gulono-1,4-lactone oxidase. Red box: upregulated in treatment with 30‰. Green box: downregulated in treatment with 30‰.

Besides GSH, L-ascorbate (AsA, also known as vitamin C) is another most abundant antioxidant in multicellular organisms [52] and is linked to glutathione metabolism [53]. Biologically, AsA is synthesized from D-galacturonate pathway through aldo-keto reductase (AKR) and L-galactono-1,4-lactone dehydrogenase (GLDH), from galactose pathway through L-galactose-1-phosphate phosphatase (GPP), L-galactose-1-dehydrogenase (GDH) and L-galactono-1,4-lactone dehydrogenase (GLDH), and from GDP-L-gulose pathway and myo-inositol pathway through L-gulono-1,4-lactone oxidase (GLO, Fig 5) [54]. In the present study, compared with the control, saline treatment downregulated expression levels of GPP and GDH, but upregulated expression levels of AKR, GLDH and GLO (S3 Table and Fig 5), suggesting that AsA might be accumulated in saline-stressed *S*. *salsa* through D-galacturonate, GDP-L-gulose and/or Myo-Inositol pathways, which might protect plants from harms of oxidation.

### Potential roles of flavonoids in adaptation to salinity

**Fig 6:**
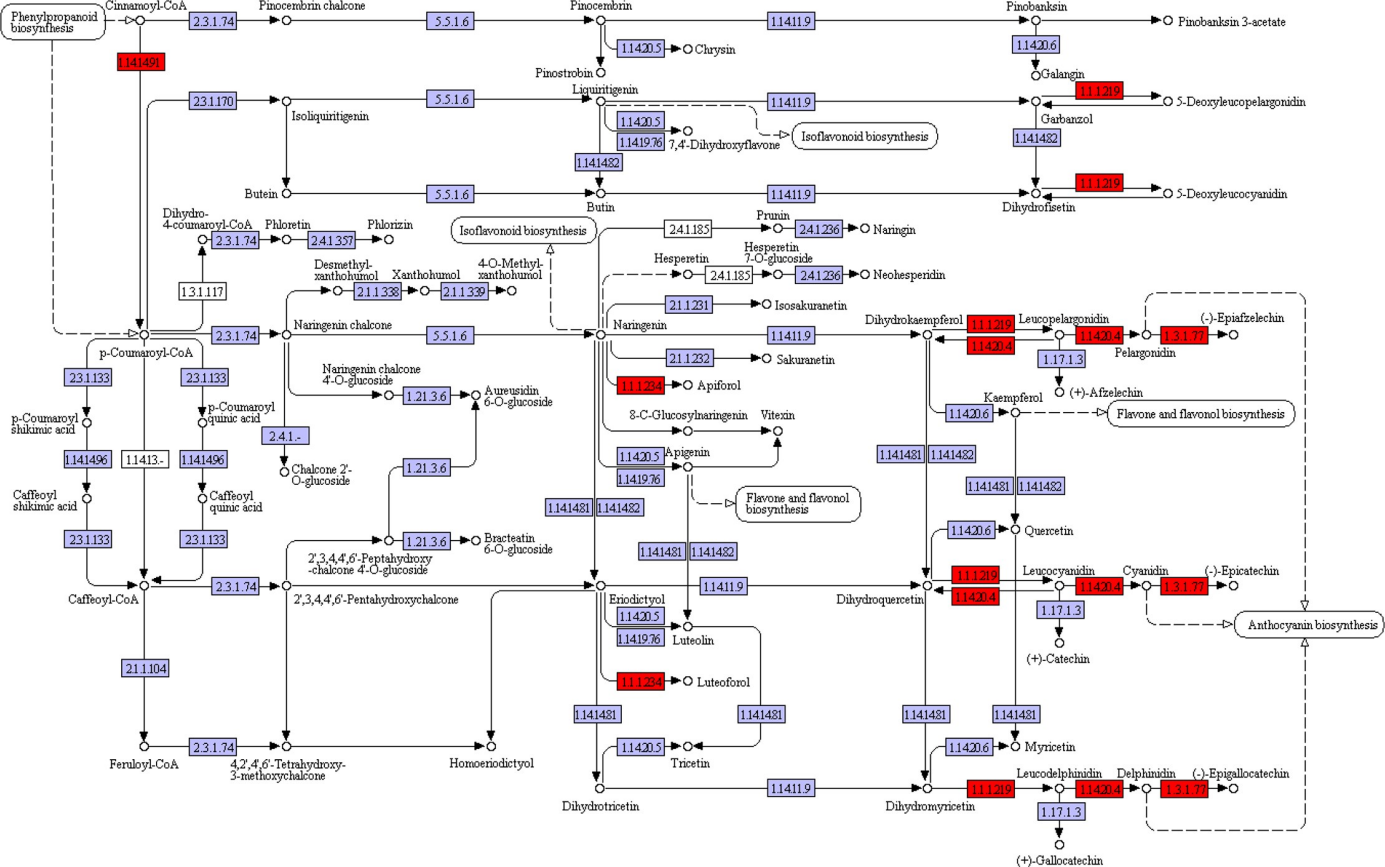
Changes of key genes in KEGG pathway of flavonoid biosynthesis. The basic flow chart was obtained from the website (https://www.kegg.jp/kegg-bin/show_pathway?map00941). Red box: significantly upregulated in treatment with 30‰ compared with the control. Purple box: no significantly change between treatment with 30‰ and the control.

Flavonoids may serve antioxidant functions in response to heavy metals, drought, and salt stresses [55–57] and modifications of flavonoid structure (i.e., glycosylation, prenylation and methylation) could inhibit lipid peroxidation in stressed plants [58,59]. In the present study, the KEGG pathway flavonoid biosynthesis (ko00941) was significantly enriched in leaves no matter upregulated or downregulated genes were subjected to KEGG enrichment analysis. Since enzymes in this pathway had lots of unigenes in the present transcriptome data, to obtain consistent results, unigenes mapped to the same enzyme were pooled and their FPKM values were summed. Finally, the results discovered six differentially expressed enzymes (Fig 6 and S4 Table), including flavonol synthase (EC 1.14.20.6), bifunctional dihydroflavonol 4-reductase/flavanone 4-reductase (EC 1.1.1.219), anthocyanidin synthase (EC 1.14.11.19), anthocyanidin reductase (EC 1.3.1.77), flavanone 4-reductase (EC 1.1.1.234) and trans-cinnamate 4-monooxygenase (EC 1.14.14.91). These results were all upregulated in response to saline treatment, indicating that flavonoid biosynthesis pathway was activated by saline treatment in *S*. *salsa* leaves.

The phenylpropanoid pathway was also upregulated in saline treatment (Table 3), which is the upstream pathway to flavonoid biosynthesis [60]. Thus, phenylpropanoids and flavonoids might function together to protect *S*. *salsa* from abiotic stresses [37, 61]. Similar results were observed in various plants, such as rice [62], wheat [63] and *S*. *glauca* [12].

### Regulation of plant hormones

**Fig 7:**
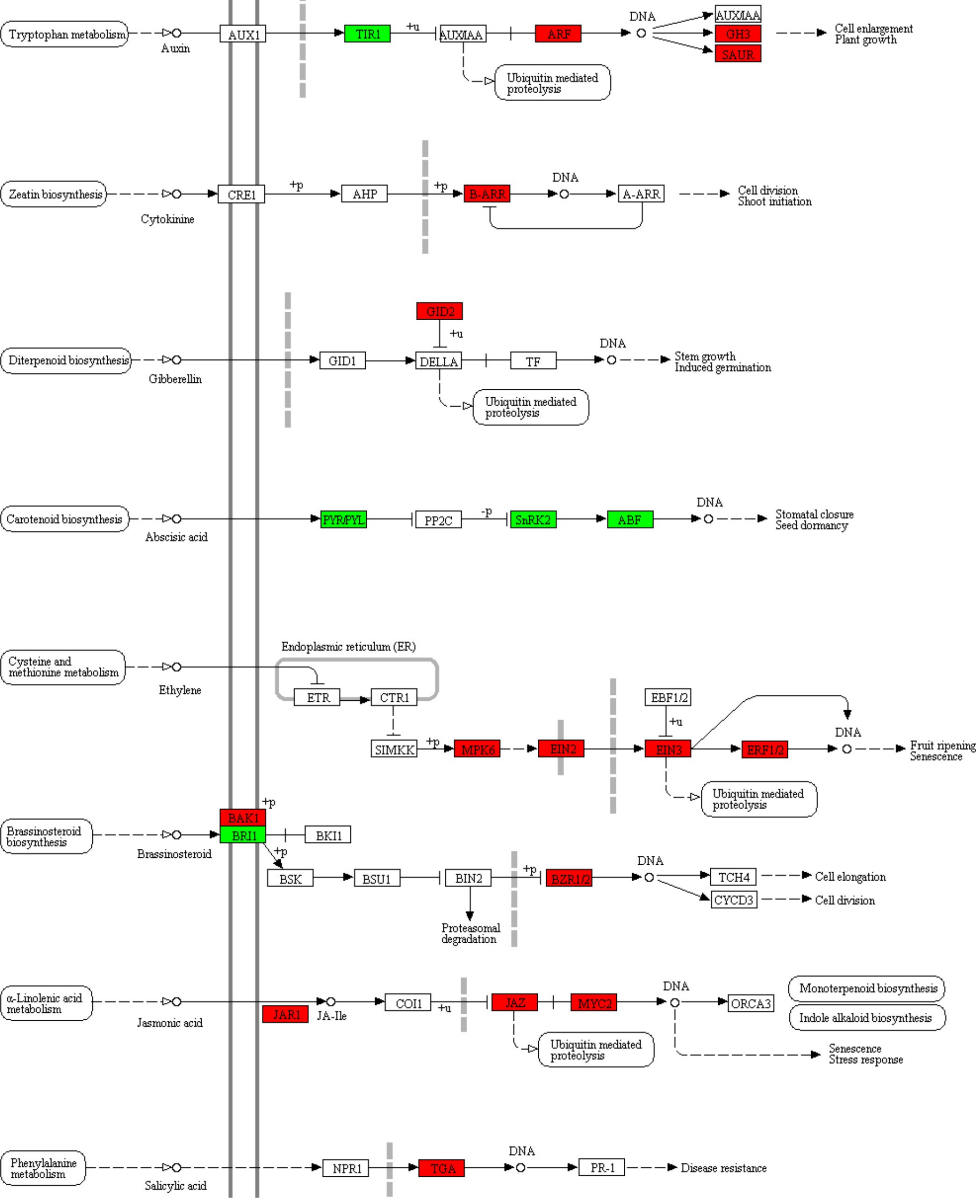
Changes of key genes in KEGG pathway of plant hormone signal transduction. The basic flow chart was obtained from the website (https://www.kegg.jp/dbget-bin/www_bget?map04075). Red box: significantly upregulated in treatment with 30‰ compared with the control. Green box: significantly downregulated in treatment with 30‰compared with the control.

The KEGG pathway plant hormone signal transduction (ko04075) is responsible to transduce signals of hormones (including auxin, cytokinine, gibberellin, abscisic acid, ethylene, brassinosteroid, jasmonic acid and salicylic acid) to downstream performance (including cell enlargement, elongation and division, germination, shoot imitation, plant growth and stress response). KEGG enrichment analyses of upregulated or downregulated genes independently both revealed significantly involvement of plant hormone signal transduction pathway in saline-treated leaves, suggesting that this pathway might play important and complicated roles in saline resistance. The FPKM values of different unigenes mapped to the same protein were summed and statistically re-analyzed by Students’ T-tests (Fig 7 and S5 Table). Only one gene in the signaling transduction of cytokinine, gibberellin and salicylic acid was upregulated. Based on the changing tendency of only one gene, it was unprecise to conclude that these three hormone signals contributed to saline resistance in *S*. *salsa*. In the brassinosteroid signaling transduction, three genes significantly differentially expressed, but their changing tendency were contradictory. Compared with the control, BAK1 was upregulated for 1.7 times, BZR1/2 was upregulated for 1.5 times, but BRI was downregulated for 1.4 times in saline treatment. These changes were quite weak and could be ignored.

Importantly, the signaling transduction of auxin, abscisic acid, ethylene and jasmonic acid involved several genes which mostly showed similar tendency in the present study, suggesting that these signaling pathways might respond to saline treatment in *S*. *salsa* and will be discussed in details.

### Potential roles of auxin signaling pathway in saline tolerance

The chemical nature of auxin is indole-3-acetic acid. In the present study, the KEGG pathway indole alkaloid biosynthesis (ko00901) was activated in saline-treated leaves. Within this pathway, the unigenes encoding methylesterase (EC 3.1.1.78) and GDSL esterase/lipase (EC 3.1.1.80) were both upregulated in saline treatment in comparison to the control (Table 5), suggesting that concentrations of indole alkaloids might be elevated. Similar results were reported previously, that indole alkaloids were induced by abiotic stresses, including UV-B irradiation [64], PEG-induced drought [65] and salinity [66]. In the auxin pathway which transduces signals of indole-3-acetic acid, auxin response factor (ARF), auxin responsive GH3 (GH3) and small auxin upregulated RNA family protein (SAUR) were also upregulated for1.7, 2.8 and 2.2 times, respectively, in saline-treated leaves (Fig 7 and S5 Table). Taken together, these results suggested that auxin might promote tolerance of *S*. *salsa* to saline stress through auxin signaling pathway.

### Changes of abscisic acid signaling in response to salinity

In the abscisic acid (ABA) signaling pathways, abscisic acid receptor (PYR/PYL), serine/threonine-protein kinase (SNRK2) and ABA responsive element binding factor (ABF) were downregulated in saline-treated leaves, compared with the control (Fig 7 and S5 Table), suggesting that ABA signaling pathway was inhibited by saline treatment. These results were different from those in non-halophytes but consistent with previous findings in a related halophyte species *S*. *maritima*. As previously reported, saline treatment increased ABA concentrations in non-halophytes tobacco [67], tomato [68], grapevine [69]. Exogenous ABA induced saline tolerant in wheat by elevating expression of peroxidase, which might reduce the active oxygen triggered by salinity [70]. Besides, ABA induced stomatal closure to minimize water loss [71]. However, in the halophyte *S*. *maritima*, saline treatments significantly decreased ABA concentration in comparison to the control [72]. Slight decrease of ABA concentration was also observed in NaCl-treated *Prosopis strombulifera* roots and leaves [73]. These results suggested that inhibition of ABA signaling might be a common strategy for halophytes to resist salinity. Jin et al. [12] also reported changes of expression levels of ABA related genes in saline treatment in *S*. *glauca*, but the authors did not explain the detailed mechanisms. The underlying mechanisms required further investigations, but one thing was clear. Decreased level of ABA signaling should ensure stomatal opening and water absorption, which are important for *S*. *salsa* growth.

### Changes of ethylene signaling in response to salinity

In *S*. *salsa* leaves, four genes responsible for ethylene signaling were significantly unregulated in saline treatment compared with the control, including mitogen-activated protein kinase (MPK6), ethylene-insensitive protein 2 (EIN2), ethylene-insensitive protein 3 (EIN3) and ethylene responsive factor 1/2 (ERF1/2) (Fig 7 and S5 Table). These results were consistent with the upregulation of ERFs in saline-treated *S*. *glauca* [12] and also in accordance with the general understanding that ethylene signaling is indispensable for tolerance to saline stress in *Arabidopsis* and many other terrestrial plants [74].

Downstream effectors of ethylene signaling could include reactive oxygen species (ROS) scavengers (such as SOD) and ion transporters [74]. In the present study, expression levels of Fe-SOD and ion transporters (including Na^+^/H^+^ exchanger, V-H^+^ ATPase, CMO, potassium channel, two-pore potassium channel and chloride channel protein) were all upregulated in response to saline treatment, which might be mediated by the activation of ethylene signaling pathway.

Moreover, ubiquitin mediated proteolysis (ko04120) is a downstream effector pathway of EIN3. It has been reported that this pathway is involved in flooding, heat, ultravioletradiation, oxidative stresses and diseases in plants [75]. In the present study, ubiquitin mediated proteolysis was upregulated in saline-stressed leaves by KEGG enrichment analysis (Table 3), suggesting that ubiquitin mediated proteolysis process might be activated to degrade salinity-denatured or oxidation-denatured proteins [76]and/or inactivate the repressors in plant hormone regulation pathway [77]. This process might be initiated by ethylene accumulation in *S*. *salsa*.

### Changes of jasmonic acid signaling in response to salinity

Jasmonic acid (JA) could enhance the tolerance of peanut [78], tomato [79]and barley [80]to saline stress by increasing antioxidant enzyme activities [78], lowering Na^+^ concentration and improving stomatal conductance [80]. In the present study, three key genes in this pathway, including jasmonic acid-amido synthetase (JAR1), jasmonate ZIM domain-containing protein (JAZ) and transcription factor MYC2 (MYC2), were significantly upregulated in saline treatment compared with the control, suggesting that activation of JA signaling might improve saline tolerance in *S*. *salsa*.

### Changes of cell wall-associated genes

In response to environmental stresses, modification of cell wall is a common defense mechanism. In both *S glauca* and *S*. *maritima*, genes related to cell wall dynamics, including laccase, expansins, leucine-rich repeat extensins (LRX), wall-associated receptor kinase proteins and/or O-acyltransferase WSD1 were upregulated in saline treatment compared with the control. In the present study, expression levels of expansins, LRX and O-acyltransferase WSD1 did not change significantly between saline treatment and the control, but laccase, and another two wall associated proteins cellulose synthase and cellulose synthase interactive [81] were significantly upregulated in the saline treatment (Table 5), suggesting that cell wall remodeling also took place in *S*. *salsa*, which might contribute to saline tolerance in *S*. *salsa* as reported in *S*. *glauca* and *S*. *maritima* [12,13].

## Conclusions

In response to treatment with 30‰ salinity, *S*. *salsa* displayed suppressed photosynthesis process, carbohydrate, lipid and amino acid metabolisms, which might inhibit growth of *S*. *salsa*. To resist saline stress, Na^+^/H^+^ exchanger, V-H^+^ ATPase, choline monooxygenase, potassium and chloride channels were upregulated to ensure ionic balance. Expression levels of Fe-SOD and genes in glutathione metabolism, biosynthesis of L-ascorbate, flavonoids and phenylpropanoids were all upregulated to produce more antioxidants, thus relieving harmful effects of saline-induced oxidation. Plant hormones played essential roles in saline resistance in *S*. *salsa*. Importantly, auxin, ethylene and jasmonic acid signaling transduction pathways were upregulated and abscisic acid signaling transduction was inhibited by saline treatment, which might activate antioxidant mechanisms and ionic adjustment.

## Supporting information

S1 Fig BUSCO analyses of unigene assembly. (TIF)

S2 Fig Length distribution of unigenes and coding sequences (CDS). (TIF)

S1 Table Primer used in the present study. (DOCX)

S2 Table Statistics of unigenes assembled based on each sample and all samples mixed together. (DOCX)

S3 Table FPKM values of key genes involved in biosynthesis of L-ascorbate. Data represent mean ± standard deviation (n = 3). * significantly different from the control (P < 0.05).
(DOCX)

S4 Table FPKM values of genes involved in flavonoid biosynthesis. Data represent mean ± standard deviation (n = 3). * significantly different from the control (P < 0.05).
(DOCX)

S5 Table FPKM values of genes involved in plant hormone signal transduction pathway. Data represent mean ± standard deviation (n = 3). * significantly different from the control (P < 0.05).
(DOCX)
