# Supplementary figures and images for: Transcriptome sequencing revealed molecular mechanisms underlying tolerance of Suaeda salsa to saline stress
Source: PLoS One. 2019 Jul 23;14(7):e0219979. doi: 10.1371/journal.pone.0219979 (PMC6650071; doi:10.1371/journal.pone.0219979)

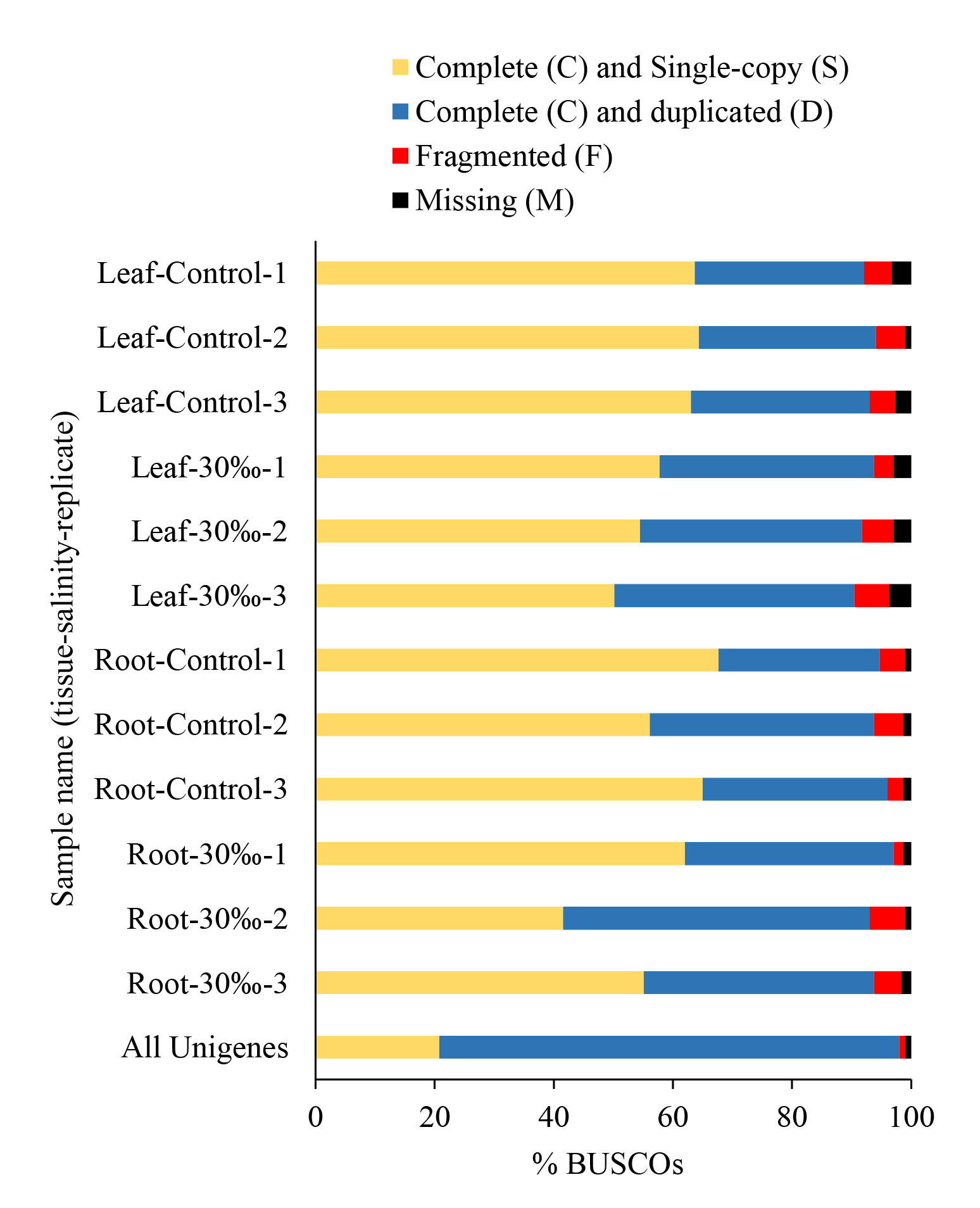

Supplement: S1 Fig — (TIF) [file pone.0219979.s001.tif]

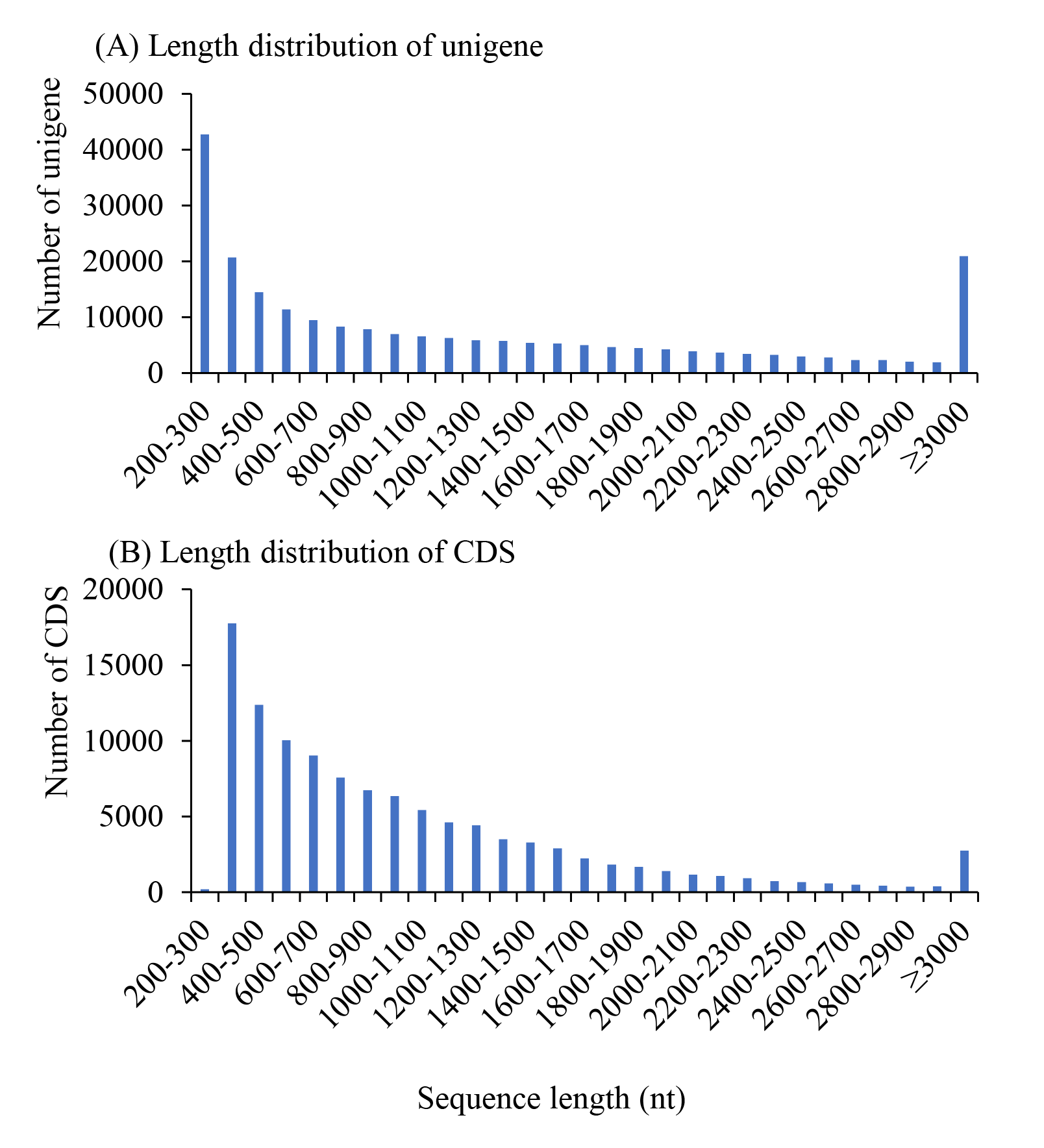

Supplement: S2 Fig — (TIF) [file pone.0219979.s002.tif]
