# Supplementary material for: Transcriptome sequencing revealed molecular mechanisms underlying tolerance of Suaeda salsa to saline stress
Source: PLoS One. 2019 Jul 23;14(7):e0219979. doi: 10.1371/journal.pone.0219979 (PMC6650071; doi:10.1371/journal.pone.0219979)
Supplement: S1 Table — (DOCX) [file pone.0219979.s003.docx]

**S1 Table. Primer used in the present study.**

| Gene name | Direction | Primer sequences |
| --- | --- | --- |
| Cationic peroxidase 1 | forward | GGCATCGTTACTTCGGTTGC |
|  | reverse | GTCAACAACCTCAAACCCGC |
| WD repeat-containing protein | forward | AGGGCAGCATCACACTTGAA |
|  | reverse | TGAAAAGGCAGCAGTGAGGG |
| 40S ribosomal protein S3a | forward | ATCTTGGCCTTGCGGATGAA |
|  | reverse | AGTGCGACATCAAGGACCTG |
| Probable linoleate 9S-lipoxygenase 5 | forward | CCAGGATTGCAGTTCAGGGT |
|  | reverse | GGCAGTGAAGGACTCGGAAA |
| Cytochrome P450 71A6 | forward | AATGTCACTTGGCGTGGCTA |
|  | reverse | AGAGGAGCATTCATGGGCAC |
| Acetyl-CoA acetyltransferas | forward | GGTCAGTGGAGAAAAGGCGA |
|  | reverse | GGGCTCCAAACCAGCATTTG |
| Lipoamide acyltransferase | forward | CCTTCACAATGTCACCCGGA |
|  | reverse | ATTGCTGAGTGCGAGCTTCT |
| Branched-chain-amino-acid aminotransferase 5 | forward | ACCGTGATTGCCAACAAACG |
|  | reverse | ACAAATTCAAGGGGGCGCTA |
| Serine/threonine-protein kinase | forward | GACAGCGGATACTTAGCGCA |
|  | reverse | CTCTGCCAGCTTCCTCCTTC |
| Beta-galactosidase 2 | forward | TTCAGACCGTATTCAGCGCA |
|  | reverse | CGCCTAGGCACTTAGTCTCG |
| Glucan endo-1,3-beta-glucosidase | forward | CCTCGTCGATAGAACCAGCC |
|  | reverse | TGGCGGCGAAGAGTTGATAA |
| Lichenase | forward | AAGCTTGCTTGTCAGACGGA |
|  | reverse | AATCAAAGGCGGTGCAATGG |
| Glucan endo-1,3-beta-glucosidase 2 | forward | TGCCGATCCTGATTTTGCCT |
|  | reverse | ACTCCCTGCGGCTTAACTTC |
| Beta-glucosidase 12 | forward | TCAAGTGCTCTCTCTGCTGC |
|  | reverse | TTACTGGGTTGCTCACCACC |
| transcriptional elongation factor-1alpha | forward | ACAAGCTTAAGGCAGAGCGT |
|  | reverse | GCACAGTCAGCTTGAGAGGT |
