# Supplementary material for: Transcriptome sequencing revealed molecular mechanisms underlying tolerance of Suaeda salsa to saline stress
Source: PLoS One. 2019 Jul 23;14(7):e0219979. doi: 10.1371/journal.pone.0219979 (PMC6650071; doi:10.1371/journal.pone.0219979)
Supplement: S2 Table — (DOCX) [file pone.0219979.s004.docx]

**S2 Table. Statistics of unigenes assembled based on each sample and all samples mixed together.**

| Salinity-replicate | TN | TL (M) | ML | N50 | N70 | N90 | GC(%) |
| --- | --- | --- | --- | --- | --- | --- | --- |
| Leaf samples | | | | | | | |
| Control-1 | 40038 | 47.27 | 1180 | 1743 | 1243 | 590 | 39.80 |
| Control-2 | 59131 | 59.17 | 1000 | 1687 | 1098 | 390 | 39.03 |
| Control-3 | 62001 | 61.67 | 994 | 1655 | 1067 | 399 | 39.45 |
| 30‰-1 | 63732 | 64.15 | 1006 | 1671 | 1079 | 407 | 38.85 |
| 30‰-2 | 77070 | 70.28 | 911 | 1525 | 927 | 362 | 38.7 |
| 30‰-3 | 84968 | 75.40 | 887 | 1448 | 865 | 363 | 38.33 |
| Root samples | | | | | | | |
| Control-1 | 45358 | 51.49 | 1135 | 1766 | 1216 | 520 | 40.09 |
| Control-2 | 90785 | 76.15 | 838 | 1342 | 777 | 345 | 38.1 |
| Control-3 | 66014 | 68.03 | 1030 | 1722 | 1118 | 414 | 39.45 |
| 30‰-1 | 52464 | 54.71 | 1042 | 1678 | 1124 | 446 | 40.81 |
| 30‰-2 | 133430 | 106.49 | 798 | 1262 | 739 | 328 | 38.45 |
| 30‰-3 | 80873 | 71.83 | 888 | 1501 | 827 | 364 | 37.86 |

TN: total number; TL: total length; ML: mean length.
