# Supplementary material for: Transcriptome sequencing revealed molecular mechanisms underlying tolerance of Suaeda salsa to saline stress
Source: PLoS One. 2019 Jul 23;14(7):e0219979. doi: 10.1371/journal.pone.0219979 (PMC6650071; doi:10.1371/journal.pone.0219979)
Supplement: S3 Table — Data represent mean ± standard deviation (n = 3). * significantly different from the control (P < 0.05). (DOCX) [file pone.0219979.s005.docx]

**S3 Table. FPKM values of key genes involved in biosynthesis of L-ascorbate.** Data represent mean ± standard deviation (n = 3). * significantly different from the control (P < 0.05).

| Gene name | Leaves | | Roots | |
| --- | --- | --- | --- | --- |
|  | Control | 30‰ | Control | 30‰ |
| GDP-L-galactose phosphorylase | 23.6±7.6 | 6.9±0.8* | 29.9±12.8 | 7.0±4.0 |
| L-galactose dehydrogenase | 7.5 ± 1.7 | 4.1±1.1* | 5.4 ± 2.8 | 3.3 ± 1.5 |
| L-galactono-1,4-lactone dehydrogenase | 1.4 ± 0.7 | 6.3±0.6* | 0.9 ± 0.8 | 3.2 ± 0.4* |
| aldo-keto reductase | 64.2±25.3 | 65.7±6.4 | 122.4±20.2 | 377.4±71.5* |
| L-gulono-1,4-lactone oxidase | 1.5 ± 0.2 | 2.4±1.0* | 4.5 ± 2.5 | 4.2 ± 3.9 |
