# Supplementary material for: Transcriptome sequencing revealed molecular mechanisms underlying tolerance of Suaeda salsa to saline stress
Source: PLoS One. 2019 Jul 23;14(7):e0219979. doi: 10.1371/journal.pone.0219979 (PMC6650071; doi:10.1371/journal.pone.0219979)
Supplement: S4 Table — Data represent mean ± standard deviation (n = 3). * significantly different from the control (P < 0.05). (DOCX) [file pone.0219979.s006.docx]

**S4 Table. FPKM values of genes involved in flavonoid biosynthesis.** Data represent mean ± standard deviation (n = 3). * significantly different from the control (P < 0.05).

| E.C. ID | Gene name | Leaves | | Roots | |
| --- | --- | --- | --- | --- | --- |
|  |  | Control | 30‰ | Control | 30‰ |
| 1.14.14.91 | Trans-cinnamate 4-monooxygenase | 1.6 ± 1.2 | 29.7±9.2* | 22.7±13.1 | 30.6 ± 6.8 |
| 2.3.1.74 | Chalcone synthase | 9.1 ± 7.8 | 52.5±58.0 | 6.7 ± 2.6 | 28.5±18.5 |
| 2.3.1.170 | Chalcone synthase | 9.1 ± 7.8 | 52.5±58.0 | 6.7 ± 2.6 | 28.5±18.5 |
| 5.5.1.6 | Vacuolar-sorting receptor | 77.8±23.5 | 42.0 ± 5.4 | 69.5±24.4 | 51.4±24.9 |
| 1.14.11.9 | Naringenin,2-oxoglutarate 3-dioxygenase | 47.3±39.0 | 128.2±63 | 18.8 ± 2.2 | 9.2 ± 3.2* |
| 1.14.20.6 | flavonol synthase | 265±52 | 806±212* | 717±216 | 501±139 |
| 1.1.1.219 | bifunctional dihydroflavonol 4-reductase/flavanone 4-reductase | 10.1 ± 3.9 | 25.7±2.5* | 5.9 ± 3.1 | 3.6 ± 1.5 |
| 1.14.11.19 | anthocyanidin synthase | 17.0 ± 4.4 | 33.8±8.5* | 51.3±24.0 | 49.6 ± 8.0 |
| 1.3.1.77 | anthocyanidin reductase | 5.7 ± 1.5 | 9.5 ± 1.4* | 1.0 ± 0.7 | 1.8 ± 0.9* |
| 1.17.1.3 | pinoresinol-lariciresinol reductase | 0.9 ± 1.6 | 0.1 ± 0.1 | 0.4 ± 0.3 | 0.4 ± 0.8 |
| 1.1.1.234 | flavanone 4-reductase | 10.1 ± 3.9 | 25.7±2.5* | 5.9 ± 3.1 | 3.6 ± 1.5 |
| 2.3.1.133 | shikimate O-hydroxycinnamoyltransferase | 104±18 | 97.6 ± 4.4 | 27.8 ± 2.5 | 19.1 ± 7.5 |
| 1.14.14.96 | 5-O-(4-coumaroyl)-D-quinate 3'-monooxygenase | 42.1±20.3 | 139±79 | 104±106 | 160±173 |
| 2.1.1.104 | caffeoyl-CoA O-methyltransferase | 1204±458 | 511±102 | 2972±460 | 2452±726 |
