# Supplementary material for: Transcriptome sequencing revealed molecular mechanisms underlying tolerance of Suaeda salsa to saline stress
Source: PLoS One. 2019 Jul 23;14(7):e0219979. doi: 10.1371/journal.pone.0219979 (PMC6650071; doi:10.1371/journal.pone.0219979)
Supplement: S5 Table — Data represent mean ± standard deviation (n = 3). * significantly different from the control (P < 0.05). (DOCX) [file pone.0219979.s007.docx]

**S5 Table. FPKM values of genes involved in plant hormone signal transduction pathway.** Data represent mean ± standard deviation (n = 3). * significantly different from the control (P < 0.05).

| Gene name | Leaves | | Roots | |
| --- | --- | --- | --- | --- |
|  | Control | 30‰ | Control | 30‰ |
| Auxin signaling transduction | | | | |
| auxin influx carrier (AUX1) | 19.9 ± 1.3 | 22.1 ± 3.1 | 39.9 ± 1.3 | 64.0 ± 10.3* |
| Protein transport inhibitor response (TRI1) | 31.6 ± 5.5 | 18.6 ± 1.2* | 27.9 ± 8.6 | 19.3 ± 4.7 |
| auxin-responsive protein IAA (AUX/IAA) | 337.3 ± 31.8 | 291.1 ± 9.2 | 347.1 ± 53.4 | 236.8 ± 42.0 |
| Auxin response factor (ARF) | 174.7 ± 28.4 | 301.1 ± 43.8* | 549.9 ± 54.7 | 395.5 ± 86.4 |
| auxin responsive GH3 (GH3) | 6.6 ± 2.7 | 18.5 ± 5.9* | 60.3 ± 44.6 | 39.4 ± 12.8 |
| SAUR family protein | 282.3 ± 23.3 | 619.7 ± 93.5* | 176.2 ± 24.6 | 153.7 ± 16.8* |
| Cytokinine signaling transduction | | | | |
| cytokinin receptor (CRE) | 5.2 ± 1.9 | 4.6 ± 1.4 | 18.3 ± 5.1 | 6.8 ± 2.6* |
| Histidine-containing phosphotransfer protein (AHP) | 3.8 ± 3.2 | 23.7 ± 16.6 | 9.7 ± 8.4 | 20.0 ± 22.2 |
| two-component response regulator ARR-B (B-ARR) | 139.9 ± 34.4 | 228.2 ± 54.7* | 192.3 ± 71.1 | 224.6 ± 122.1 |
| Two-component response regulator ORR (A-ARR) | 49.6 ± 15.4 | 27.9 ± 5.5 | 113.9 ± 10.1 | 33.3 ± 2.9* |
| Gibberellin signaling transduction | | | | |
| release factor glutamine methyltransferase (GID2) | 0.1 ± 0.1 | 2.4 ± 0.2* | 0.9 ± 0.8 | 3.8 ± 1.8 |
| gibberellin receptor (GID1) | 29.1 ± 14.6 | 43.6 ± 5.8 | 242.5 ± 102.9 | 81.7 ± 19.5 |
| Scarecrow-like protein (DELLA) | 201.5 ± 30.1 | 127.2 ± 22.9 | 354.0 ± 106.1 | 208.4 ± 18.6 |
| phytochrome-interacting factor (TF) | 152.5 ± 19.4 | 144.3 ± 12.8 | 129.7 ± 20.2 | 131.3 ± 18.1 |
| Abscisic acid signaling transduction | | | | |
| abscisic acid receptor PYR/PYL | 110.9 ± 14.3 | 72.4 ± 9.1* | 183.4 ± 72.1 | 157.9 ± 41.0 |
| protein phosphatase 2C (PP2C) | 35.4 ± 5.4 | 45.5 ± 6.7 | 33.9 ± 2.5 | 33.6 ± 19.1 |
| serine/threonine-protein kinase SRK2 (SNRK2) | 411.7±124.9 | 167.8 ± 24.7* | 434.6 ± 102.2 | 141.5 ± 44.5* |
| ABA responsive element binding factor (ABF) | 126.7 ± 8.8 | 51.9 ± 0.6* | 87.9 ± 17.5 | 63.6 ± 11.9 |
| Ethylene signaling transduction | | | | |
| ethylene receptor (ETR) | 22.7 ± 6.5 | 24.8 ± 7.0 | 16.6 ± 4.3 | 19.6 ± 6.0 |
| /serine/threonine-protein kinase CTR1 (CTR1) | 32.1 ± 9.5 | 43.9 ± 3.3 | 24.9 ± 11.8 | 72.5 ± 40.4 |
| mitogen-activated protein kinase kinase (SIMKK) | 6.0 ± 1.7 | 1.9 ± 0.8 | 36.2 ± 7.8 | 73.6 ± 15.1* |
| mitogen-activated protein kinase (MPK6) | 1.0 ± 0.6 | 2.6 ± 1.2* | 2.1 ± 1.7 | 2.5 ± 2.4 |
| thylene-insensitive protein 2 (EIN2) | 18.5 ± 20.1 | 43.8 ± 18.9* | 37.8 ± 38.0 | 62.1 ± 75.3 |
| EIN3-binding F-box protein (EBF1/2) | 35.8 ± 12.2 | 161.2 ± 34.2* | 48.2 ± 9.0 | 134.9 ± 81.6 |
| thylene-insensitive protein 3 (EIN3) | 0.8 ± 0.2 | 5.3 ± 0.3* | 16.3 ± 7.6 | 10.1 ± 2.9 |
| ethylene responsive factor (ERF1/2) | 47.2 ± 7.2 | 161.6 ± 23.7* | 87.9 ± 30.5 | 93.3 ± 27.6 |
| Brassinosteroid signaling transduction | | | | |
| brassinosteroid insensitive 1 (BAK1) | 8.3 ± 3.5 | 14.2 ± 4.7* | 12.8 ± 8.1 | 17.6 ± 13.0 |
| protein brassinosteroid insensitive 1 (BRI1) | 240.1 ± 24.6 | 172.3 ± 17.0* | 180.9 ± 17.6 | 161.0 ± 8.7 |
| BRI1 kinase inhibitor (BKI1) | 4.5 ± 4.8 | 1.5 ± 1.7 | 0.2 ± 0.3 | 0.0 ± 0.0 |
| BR-signaling kinase (BSK) | 25.1 ± 9.1 | 24.6 ± 5.7 | 45.8 ± 11.7 | 46.1 ± 17.0 |
| protein brassinosteroid insensitive 2 (BIN2) | 29.9 ± 16.3 | 11.5 ± 2.1 | 35.8 ± 39.2 | 16.9 ± 5.8 |
| brassinosteroid resistant 1/2 (BZR1/2) | 69.2 ± 2.5 | 101.5 ± 7.8* | 68.9 ± 4.9 | 79.1 ± 26.4 |
| xyloglucan:xyloglucosyl transferase (TCH4) | 353.1 ± 99.1 | 285.5 ± 9.3 | 298.8 ± 61.4 | 615.2 ± 311.1 |
| Cyclin-D3 (CYCD3) | 8.3 ± 6.1 | 2.3 ± 0.8 | 6.5 ± 2.0 | 9.3 ± 2.0 |
| Jasmonic acid signaling transduction | | | | |
| Jasmonic acid-amido synthetase (JAR1) | 21.4 ± 7.1 | 52.1 ± 7.4* | 49.9 ± 19.3 | 18.8 ± 5.7* |
| Coronatine-insensitive protein 1 (COI1) | 10.4 ± 2.0 | 4.3 ± 1.1 | 9.8 ± 5.6 | 9.3 ± 6.3 |
| jasmonate ZIM domain-containing protein (JAZ) | 155.2 ± 62.8 | 1222.6±333.0* | 617.2 ± 286.0 | 930.7 ± 574.3 |
| transcription factor MYC2 (MYC2) | 83.0 ± 6.0 | 165.8 ± 22.6* | 124.3 ± 21.0 | 107.9 ± 28.6 |
| Salicylic acid signaling transduction | | | | |
| Regulatory protein 5 (NPR5) | 22.7 ± 6.7 | 9.3 ± 2.4 | 27.4 ± 2.9 | 33.8 ± 10.6 |
| Transcription factor (TGA) | 47.5 ± 3.8 | 85.4 ± 12.0* | 62.9 ± 13.4 | 50.4 ± 29.4 |
| pathogenesis-related protein 1 (PR-1) | 237.8 ± 239.0 | 328.0 ± 313.2 | 1156.7 ± 337.4 | 2072.4 ± 858.0 |
